# Unusual Roles of Discharge, Slope and SOC in DOC Transport in Small Mountainous Rivers, Taiwan

**DOI:** 10.1038/s41598-018-38276-x

**Published:** 2019-02-07

**Authors:** Li-Chin Lee, Ting-Chang Hsu, Tsung-Yu Lee, Yu-Ting Shih, Chuan-Yao Lin, Shih-Hao Jien, Thomas Hein, Franz Zehetner, Fuh-Kwo Shiah, Jr-Chuan Huang

**Affiliations:** 1 Department of Geography, National Taiwan University, Taipei, Taiwan; 2 Department of Geography, National Taiwan Normal University, Taipei, Taiwan; 3 Research Center for Environmental Changes, Academia Sinica, Taipei, Taiwan; 4 Department of Soil and Water Conservation, National Ping Tung University of Science and Technology, Ping Tung, Taiwan; 5 Department of Water, Atmosphere and Environment, Institute of Hydrobiology and Aquatic Ecosystem Management, University of Natural Resources and Life Sciences, Vienna, Austria; 6 Department of Forest and Soil Sciences, Institute of Soil Research, University of Natural Resources and Life Sciences, Vienna, Austria

## Abstract

Riverine dissolved organic carbon (DOC), responsible for riverine productivity, is rarely documented in subtropical small mountainous rivers (SMRs) where high rainfall and steep slopes are the main features. This study investigated the DOC export at eight sites in three Taiwan SMRs to characterize the dynamics and controlling factors of DOC transport. Results showed that the mean DOC concentration of ~0.78 mg L^−1^ is much lower than the global average of ~5.29 mg L^−1^. However, the mean DOC yield, ~22.51 kg-C ha^−1^ yr^−1^, is higher than the global average of 14.4–19.3 kg-C ha^−1^ yr^−1^. Comparing with worldwide rivers from literature, the annual discharge, slope, and SOC (soil organic carbon) are controlling factors as expected, though they influence in different ways. SOC stock likely regulated by elevation-dependent biomes dominate the DOC supply, while slope restrains the DOC generation due to shallow soil depth and fast runoff velocity. However, the abundant discharge flushing this persistent low supply leads to a large DOC export in the SMRs. Furthermore, the DOC dynamics during typhoon periods showed a clockwise hysteresis, suggesting that the DOC is mainly from the riparian zone or downslope area during the rising limb of the hydrograph. This study elucidates the DOC transport in SMRs and provides an atypical yet significant piece of understanding on DOC transport in a global context.

## Introduction

Riverine dissolved organic carbon (DOC), which serves as the major energy source for aquatic microbial communities, linking different carbon pools, interacting with heavy metals and attenuating UV radiation, is governed by the abiotic and biotic biogeochemical processes^1–5^. Globally, exorheic rivers transport ~0.17–0.22 Pg-C yr^−1^ of terrestrial DOC to the ocean, with the mean concentration at 5.29 mg-C L^−1^^6–8^. And the riverine DOC export increases with rising primary productivity from the temperate to the tropical zone. For examples, the Arctic rivers which account for 14.9% of the global land can contribute ~12% of the global riverine DOC^9^ and rivers between 30°N and 30°S, covering ~42.7% of the global land area, export 55–62% of the global riverine DOC with the mean concentration at 5.38 mg-C L^−1^^8–10^. This disproportional export in low latitude indicates the importance of the tropical/ subtropical regions in terrestrial-ocean DOC transport. In these regions, SMRs (small mountainous rivers) have been hypothesized to have a high DOC export and most of them flow directly and rapidly to the ocean^11^. However, the dynamic variation and associated factors of DOC export in SMRs are far from well-understood.

The hydroclimate, biome, and landscape are well-recognized controlling factors in regulating riverine DOC export, since those factors alter microbial communities and biogeochemical processes by ways of physical, chemical, and biological functioning. Previous studies have applied those factors to estimate DOC export on watershed, regional or global scales. For examples, Ludwig, *et al*.^12^ compiled the DOC export of 29 main rivers around the world and concluded the mean annual runoff, basin slope, and the soil organic carbon (SOC) are controlling factors. Aitkenhead-Peterson and McDowell^13^ compiled the annual DOC fluxes from 164 watersheds and classified those watersheds into 15 biomes and then found that the mean soil C/N ratio, highly associated with biome, is also a good predictor to estimate annual DOC fluxes on local and global scales. Hope, *et al*.^14^ concluded that annual precipitation and SOC could explain 94% of the variation in riverine DOC exports through 17 British rivers. Although the streamflow (or precipitation), slope gradient, and SOC (or biome) are good predictors for DOC export on catchment scale, the effectiveness of the factors in SMRs are less discussed, which can improve the understanding of DOC export in subtropical SMRs.

Among the controlling factors, runoff which flows through land surface and subsurface and connects the hillslope to stream, has a unique role in transporting DOC^15,16^. Therefore, the flushing hypothesis which affirms that the terrestrial C accumulates in the riparian zone and near-stream hillslopes at normal flow regime are subsequently flushed by storms as the water table rises is proposed^17^. This hypothesis highlights the alternating nature of DOC export during rainstorms. In general, typhoons (alias of tropical cyclone in the West Pacific) usually bring torrential rainfall within 48–72 hr, resulting in massive substrate transport through rivers in Taiwan^18,19^. Lee, *et al*.^20^ indicated that ~25% of the annual DOC flux was contributed by typhoon events in a Taiwan SMR. However, the flushing hypothesis and the DOC transport behaviors during rainstorms in different regions are rarely compared and discussed. Understanding the DOC transport behaviors during rainstorms (e.g. typhoon) can advance the knowledge on the alternating nature of DOC transport.

In this study, we investigated the DOC export behaviors at eight sites in three SMRs with different environmental backgrounds. The LOADEST (Load Estimator) developed by Runkel, *et al*.^21^ was applied for DOC flux estimation. Based on this dataset, we tried to determine: (1) the DOC concentrations and fluxes in the three SMRs; (2) differences of controlling factors in SMRs and other large rivers and (3) the flushing behavior of DOC transport during typhoon events. Through investigating the interaction among SOC, slope, and discharge on DOC export, the unique DOC transport in the subtropical SMRs will be better understood and potentially improve the assessment of the global carbon cycle.

## Background Of Studied SMRs

Taiwan has a unique locality at the juncture between the Philippine and Eurasian tectonic plates and in the corridor of tropical cyclones^18,22^. Steep and fractured landscape accommodating frequent typhoon invasions is destined to be Taiwan’s geographical characteristics. Specifically, the dramatically uplifting elevation (0–4,000 m) within a short horizontal distance (~75 km) results in fast streamflow velocity. Climatically, the precipitation in wet season, from May to October, accounts for 70% of the annual precipitation. The convective rainstorms and typhoons are the main contributors. On the other hand, the precipitation in dry season, November to April of the following year, is diminutive and gets scarcer from northern to southern Taiwan. Corresponding to precipitation, the discharge in the dry season is usually quite low and stable, whereas in the wet season discharge triggered by typhoons (3–5 times a year) is periodic and spiky. Herein we collected a large amount of DOC data in three Taiwan SMRs during normal flow regime and typhoon events.

**Figure 1:**
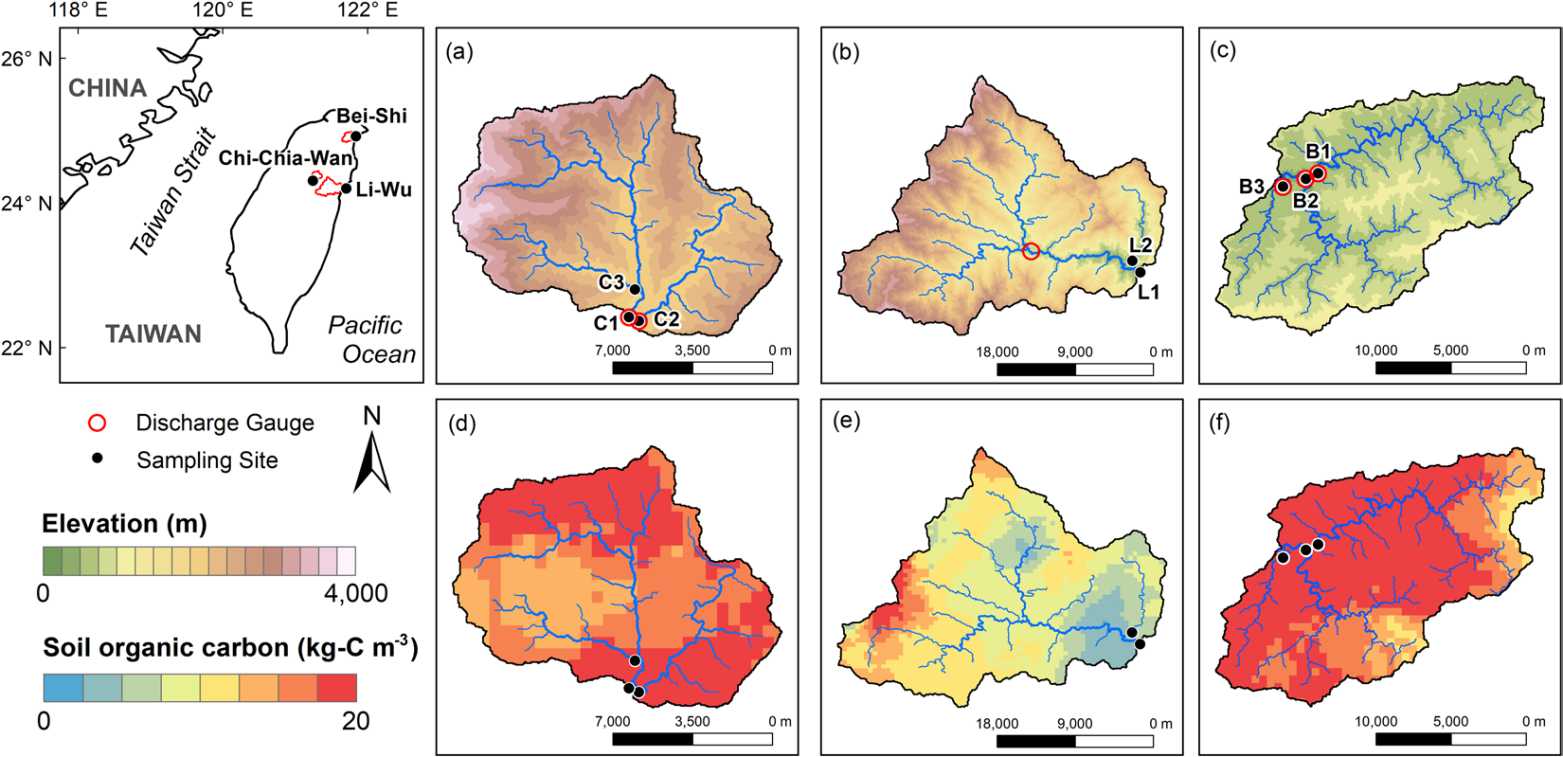
Locations of the sampling sites in Chi-Chia-Wan, Li-Wu and Bei-Shi River. The elevation distribution of Chi-Chia-Wan (**a**), Li-Wu (**b**) and Bei-Shi (**c**) River. Spatial SOC distribution of Chi-Chia-Wan (**d**), Li-Wu (**e**) and Bei-Shi (**f**) River.

The first river is Chi-Chia-Wan River, the renowned sole habitat for the endangered landlocked salmon, *Oncorhynchus masou formosanus*^22^. In this river, three sampling sites were selected at C1, C2, and C3 due to different land use configurations. The second one is Li-Wu River, which drains through the marble stratum and carves out Taroko Gorge. Due to the landscape significance of Taroko Gorge, Li-Wu River is a part of the Taroko National Park and hence, apart from tourism, all human activities are strictly prohibited^18^. Two sampling sites at L1 and L2 were set to represent the entire Li-Wu River and the main tributary. The last river is Bei-Shi River whose sampling sites were set at B1, B2, and B3 tributaries. These three tributaries confluence in Pin-Lin and then flow into Fei-Tsui Reservoir, which is the main domestic water supply for Taipei metropolis^23,24^. The sampling locations of the studied SMRs were shown in Fig. 1. Additionally, the detailed hydroclimatic metrics, landscape setting, sampling scheme, and the method of DOC measurement were described in the supplementary material.

## DOC Concentrations and Yields

**Figure 2:**
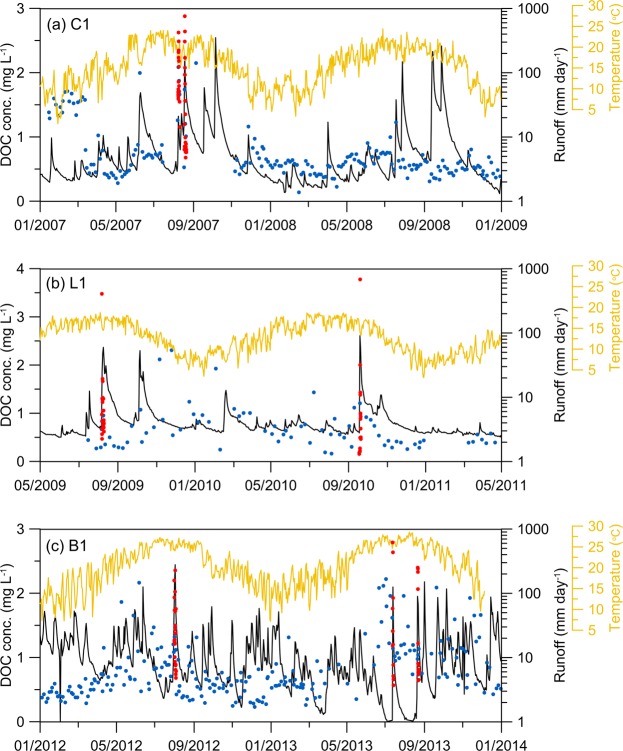
The discharge, air temperature, and DOC concentration at C1 during Jan. 2007-Dec. 2008 (**a**), L1 during May 2009-Apr. 2011 (**b**) and B1 during Jan. 2012-Dec. 2013 (**c**). The red and blue dots represent the observed DOC concentration during typhoon and non-typhoon period (corresponding to left *y*-axis). The black and yellow lines indicate the runoff and temperature (corresponding to right *y*-axes).

The chronosequence of air temperature, runoff, and DOC concentrations at C1, L1, and B1 were shown as examples in Fig. 2. The mean annual temperature of the two adjacent sites C1 and L1 were 10 °C and 13 °C, respectively. The obvious daily and seasonal variations of temperature could be found at C1, because the C1 site is located between the high standing Central Range and the Snow Mountain Range where a large portion of sunlight is blocked. The B1 site located in lower elevation, as expected, has the highest annual mean temperature of 19 °C and distinct daily and seasonal variations. Regarding runoff, the seasonal variations at C1 and L1 were larger than that at B1, since the rainfall seasonality at B1 is indistinct due to the gentle rainfall brought by the winter monsoon. Meanwhile, in the summer, the surged runoff induced by typhoons and rainstorms is notably high, around 2-order of magnitude higher than the normal flow regime. The DOC concentrations at C1 and L1 sites were quite low (<1.0 mg L^−1^) but were elevated during typhoon periods. Although the DOC concentrations at B1 were relatively high during the sampling period, most of them were less than 2.0 mg L^−1^.

**Figure 3:**
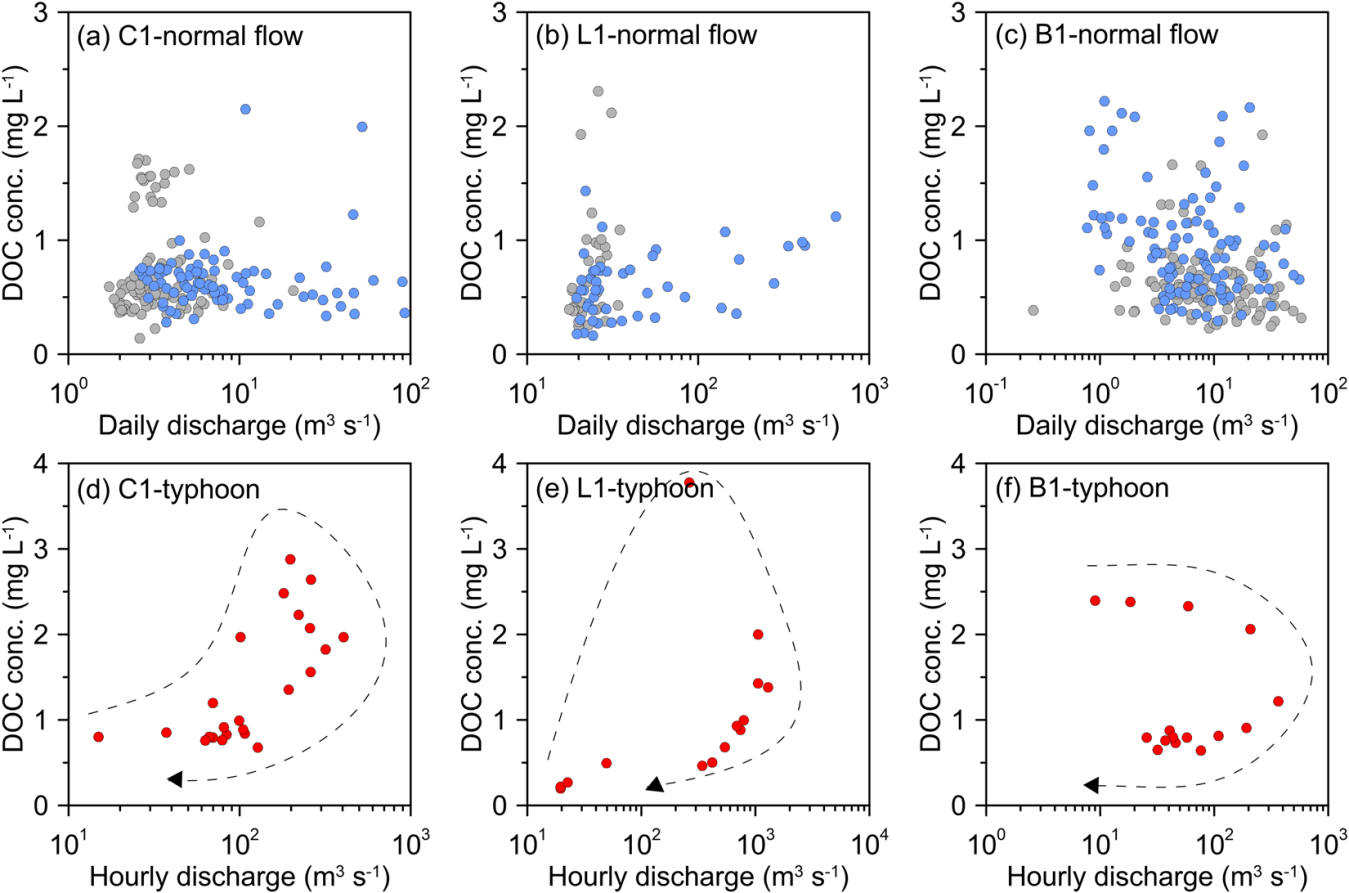
The relationship between DOC concentration and discharge in normal flow regime at C1 (**a**), L1 (**b**), and B1 (**c**). The gray and blue dots indicate samples during the dry and wet season, respectively. The C-Q relationship during the selected typhoon events at C1 (**d**), L1 (**e**), and B1 (**f**). The clockwise curve with an arrow indicates the time sequence of a typhoon period.

The C-Q relationships (DOC concentration vs discharge) at C1, L1, and B1 were shown in Fig. 3. It shows that the DOC concentrations during normal flow regime were irrelative with discharge and had no distinct seasonality, even though the SMRs are located in the warm subtropical. Only a little negative C-Q relation could be found at B1, but without statistical significance. Comparing to other rivers, Raymond, *et al*.^25^ demonstrated that, in Arctic rivers, the DOC concentration is significantly correlated to water discharge. Large export of DOC was found during the spring thaw. Conversely, in Mississippi River, the DOC concentration showed small temporal variability throughout the year^26^. As for typhoon events, the DOC concentration showed a significant clockwise hysteresis (Fig. 3d–f). The concentrations increased rapidly during the rising limb of the hydrograph and reached the apex before the peak flow. The DOC concentrations of the tropical SMR in Guadeloupe were positively correlated to discharge with a little clockwise hysteresis^11^. In southeast Alaska, the DOC concentration simultaneously echoed with discharge during rainstorms^27^. However, our unsynchronized C-Q relationships imply that the limited DOC storage is easily flushed and then exhausted. Thus, the DOC concentration drops quickly as peak flow comes, which could be attributed to the flushing hypothesis^17^.

The mean annual DOC concentrations, runoff and DOC yields of the eight sites were shown in Table 1. Generally, the mean annual DOC concentrations of all sites were approximately 0.78 mg L^−1^ and the mean seasonal concentrations were 0.74 and 0.82 mg L^−1^ for the dry and wet season, respectively. Bei-Shi River was the only one of three rivers that showed a little seasonal variation, whereas no significant seasonal variation could be detected in the other two rivers. In Chi-Chia-Wan River, the DOC concentration at C2 was higher than that at C1 and C3, probably due to a little higher proportion of agricultural land (Fig. S1). The annual mean daily runoff of the eight sites were 7.88 mm d^−1^ (equivalent to 2,875 mm per year) and the mean seasonal runoff ranged between 4.99 and 10.76 mm d^−1^ for the dry and wet season. In comparison, the annual runoff in Li-Wu was much lower than the other two rivers. The seasonal variation in Chi-Chia-Wan River was much more evident than the other two rivers. The average daily DOC yield of all sites was 61.61 g-C ha^−1^ d^−1^, equivalent to 22.51 kg-C ha^−1^ yr^−1^, with the range of 30.48 to 78.31 g-C ha^−1^ d^−1^. The estimated DOC yields in Li-Wu River were 50% lower (30.48 and 34.22 g-C-ha^−1^ d^−1^ at L1 and L2, respectively) than the other two rivers (overall, ~70 g-C ha^−1^ d^−1^, except B3 in Bei-Shi River at 51.39 g-C ha^−1^ d^−1^). For the dry season, most of the average daily DOC yields were ~20–30 g-C ha^−1^ d^−1^, but the yields at B1 and B2 in Bei-Shi River were ~2-fold higher than the others. For the wet season, the daily DOC yields for all sites in the Chi-Cha-Wan and Bei-Shi Rivers ranged from 69.04–135.29 g-C ha^−1^ d^−1^; however, the daily DOC yields for the two sites in Li-Wu Rivers were only ~40 g-C ha^−1^ d^−1^.

**Table 1:** Mean annual DOC concentrations/yields and the environmental metrics of each sampling sites.

Site	DOC conc. (mg L^−1^)	DOC yield (kg-C ha^−1^ yr^−1^)	Area (km^2^)	Mean annual Temp. (°C)	Mean annual runoff (mm yr^−1^)	Mean daily discharge (m^3^ s^−1^)	Mean surface slope (radian)	SOC (kg-C m^−3^)
C1	0.71 ± 0.17	28.62 ± 9.46	105.0	10.3 ± 0.2	3,447 ± 24	11.47 ± 29.39	0.59 ± 0.18	19.6 ± 1.5
C2	0.97 ± 0.30	26.41 ± 9.31	31.0	11.8 ± 0.2	2,628 ± 24	2.58 ± 8.67	0.52 ± 0.16	20.2 ± 0.1
C3	0.67 ± 0.15	25.71 ± 5.03	21.1	10.1 ± 0.2	3,544 ± 23	2.36 ± 5.63	0.64 ± 0.17	18.3 ± 1.3
L1	0.70 ± 0.17	11.13 ± 3.59	607.4	13.1 ± 0.4	1,737 ± 6	33.45 ± 42.83	0.66 ± 0.19	13.6 ± 2.4
L2	0.78 ± 0.20	12.49 ± 1.74	61.6	14.9 ± 0.4	1,737 ± 6	3.39 ± 4.34	0.70 ± 0.20	11.7 ± 0.9
B1	0.79 ± 0.25	27.96 ± 0.85	110.4	19.4 ± 0.6	3,782 ± 14	13.22 ± 18.73	0.39 ± 0.16	21.3 ± 2.4
B2	0.78 ± 0.19	29.02 ± 2.18	78.8	19.0 ± 1.0	3,835 ± 18	9.57 ± ± 16.53	0.45 ± 0.15	20.0 ± 1.9
B3	0.83 ± 0.19	18.78 ± 2.90	22.4	19.6 ± 1.0	2,340 ± 15	1.66 ± 3.96	0.42 ± 0.14	22.3 ± 0.8
Average	0.78 ± 0.09	22.51 ± 7.36	129.7	14.8 ± 4.1	2,881 ± 882	9.71 ± 10.61	0.55 ± 0.12	18.4 ± 3.8

^*^Mean value ± one standard deviation.

## DOC Concentrations In SMRs and Other Rivers

Generally, the riverine DOC concentration mostly varies between 1.0 to 20.0 mg L^−1^ worldwide^6^ with a mean value of 5.29 mg L^−1 ^^8^. On a regional scale, the concentrations of DOC in the wet tropical and temperate zone are observed to be approximately 6.0 and 3.0 mg L^−1^^6^, respectively. However, our mean DOC concentrations (~0.78 mg L^−1^, with the range of 0.67 to 0.97 mg L^−1^ in Table 1) are considerably lower than the reported values in the literature. Lower DOC concentrations were likely attributed to steeper landscape characteristics. For example, Alin, *et al*.^28^ showed that, in Papua New Guinea, the slope in Strickland is steeper than in Fly River, and the DOC concentrations in Strickland and Fly are 2.53 and 3.85 mg L^−1^, respectively. In New Zealand, the DOC concentrations in the North Island and South Alpine are ~2.39 and ~0.38 mg L^−1 ^^29^, respectively. The streams draining the steep Southern Alps in South Island also result in lower DOC concentration, though other factors (e.g. glacier cover) may alter the DOC production as well. Also, the Pearl River in South China, where climate is similar to Taiwan, though with gentle landscape and large drainage area, the DOC concentration is as low as ~1.5 mg L^−1 ^^30^. In the subtropical mountainous watersheds of Wuyi, China, where latitude, precipitation, SOC and soil C/N ratio are similar to Taiwan except slope, the DOC concentrations in these watersheds of Wuyi were ~4.80 mg L^−1 ^^31^, ~5-fold higher than that in Taiwan. Collectively, steeper slope that leads to higher flow velocity and shorter residence time impedes DOC accumulation.

## DOC Yields In Subtropical SMRs and Other Rivers

**Figure 4:**
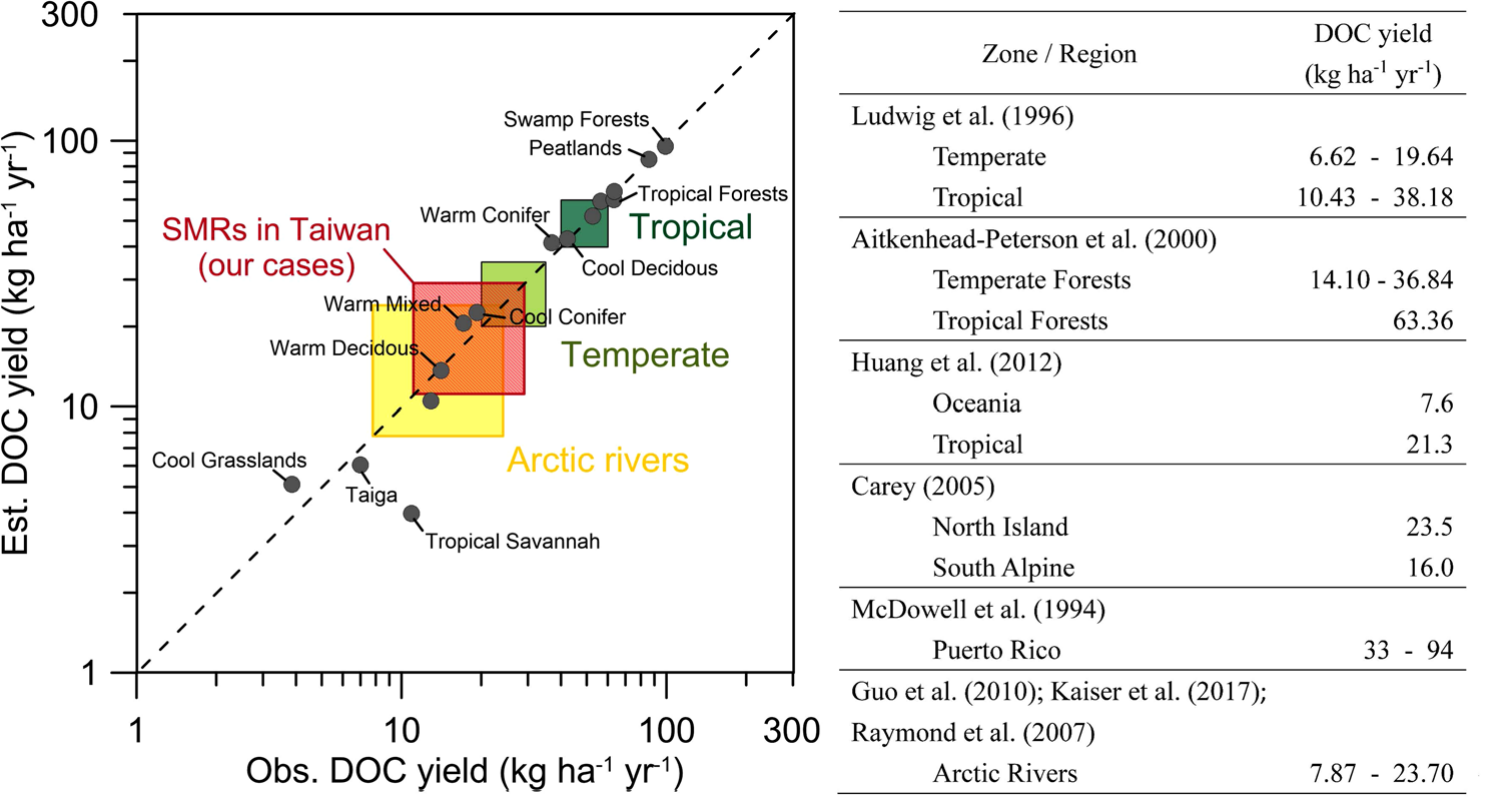
The DOC yield in different biomes and regions from the literature and Taiwan SMRs (red box). The gray dots derived from Aitkenhead-Peterson and McDowell^13^ show the observation and estimation of DOC yields in 15 biome types on a global scale. The light and dark green box indicate the approximation of DOC yields in temperate and tropical forests (referring to section 5). The yellow box shows the DOC yields in Arctic rivers.

The average annual DOC yield of worldwide rivers is ~14.4–19.3 kg-C ha^−1^ yr^−1 ^^6,8,12^ which is highly associated with the physio-geographic factors. Ludwig, *et al*.^12^ concluded that the DOC yields for the temperate forests are 6.6–19.6 kg-C ha^−1^ yr^−1^, and 10.4–38.2 kg-C ha^−1^ yr^−1^ for the tropical forests (Fig. 4). Similarly, Aitkenhead-Peterson and McDowell^13^ compiled 164 rivers, and classified the DOC yields into 15 biome types based on Meybeck^6^, and they obtained DOC yields of 14.1–36.8 in warm forests and 63.3 kg-C ha^−1^ yr^−1^ in the tropical forests, which are higher than the estimation of Ludwig, *et al*.^12^. Despite the inconsistence between the two estimations in tropical and temperate forests, the DOC flux in the tropics is consistently larger than that in the temperate zones. Additionally, other isolated local studies published that the DOC yield in tropical forests of Puerto Rico is between 33–94 kg-C ha^−1^ yr^−1 ^^32^ and 23.5 kg-C ha^−1^ yr^−1^ in the temperate forests of North Island of New Zealand and 16.0 kg-C ha^−1^ yr^−1^ in the South Alpine of New Zealand^29^. Furthermore, the Arctic river watersheds, mostly covered with tundra and taiga, contribute ~12% of DOC flux to the ocean^9^ and the DOC yields of Arctic rivers are documented around 7.9 to 23.7 kg-C ha^−1^ yr^−1 ^^25,33,34^. Compiled those pieces as a whole, the DOC yields decrease from tropical to temperate, and to taiga and tundra, following the latitude gradient. The approximate DOC yield from tundra and taiga, to temperate and to tropical forests are between 8.0–24, 20–35 and 40–60 kg-C ha^−1^ yr^−1^, respectively (Fig. 4).

Comparatively, the DOC yields in our cases (11.1~29.0 kg-C ha^−1^ yr^−1^) are substantially low than previous estimates from the tropical forests, a little higher than that in Arctic rivers, but similar to the range between warm deciduous (14.1 kg-C ha^−1^ yr^−1^) and warm conifer forests (36.8 kg-C ha^−1^ yr^−1^)^13^. In fact, the biome in the mountainous region changes from subtropical broadleaf to warm deciduous broadleaf, and to cold temperate coniferous along the increase of elevation. Most parts of the 3 SMRs are occupied by the cool temperate mixed broadleaf and coniferous forest. Therefore, when estimating the DOC export in mountainous region, the elevation-dependent biome should be taken into account, rather than latitude alone.

## Controlling Factors of DOC Estimations In SMRs

For estimating DOC export, a multiple regressive model with variables of annual runoff, mean slopes, and SOC was proposed to estimate the DOC yields^12^. Note that a correlation coefficient matrix (including additional variables of population, agriculture and temperature) was presented in the supplementary material (Table S3). That correlation coefficients affirm only three variables, namely, annual runoff, mean slopes, and SOC, play a dominant role in DOC export. If we apply Ludwig’s regressive model onto Taiwan datasets (Table 1), the DOC yields should be 104.7, 24.8, and 132.87 kg-C ha^−1^ yr^−1^ at C1, L1, and B1 which are 2 to 5-fold higher than our observations at 28.6, 11.1, and 28.0 kg-C ha^−1^ yr^−1^. Despite the large overestimation, that a high correlation between estimations and observations was held (correlation coefficient = 0.93) indicates the effectiveness of the variables.

We further used our dataset on Aitkenhead-Peterson and McDowell^13^ model that used soil C/N ratio as a dominant predictor for DOC yield. In Taiwan, the average SOC stock and soil C/N ratio are approximately 10–20 kg-C m^−3^ (see Table 1) and ~7–15^35^. Our SOC is similar to other forested mountainous regions; however, the soil C/N ratio is a little low in comparison with other systems worldwide indicating the N-enriched condition, likely due to the high N deposition, over 2000 kg-N km^−2 ^^24^ and high mineralization of organic carbon in the acid soil^36^. Following Aitkenhead’s regressive model, if the soil C/N ratio of Taiwan from Chen, *et al*.^35^ is applied, the estimated DOC yield will be less than 10 kg-C ha^−1^ yr^−1^, which is much lower than our observations. The large discrepancy between our observations with the two estimations through Ludwig and Aitkenhead’s work manifested the unusual DOC export in SMRs.

**Figure 5:**
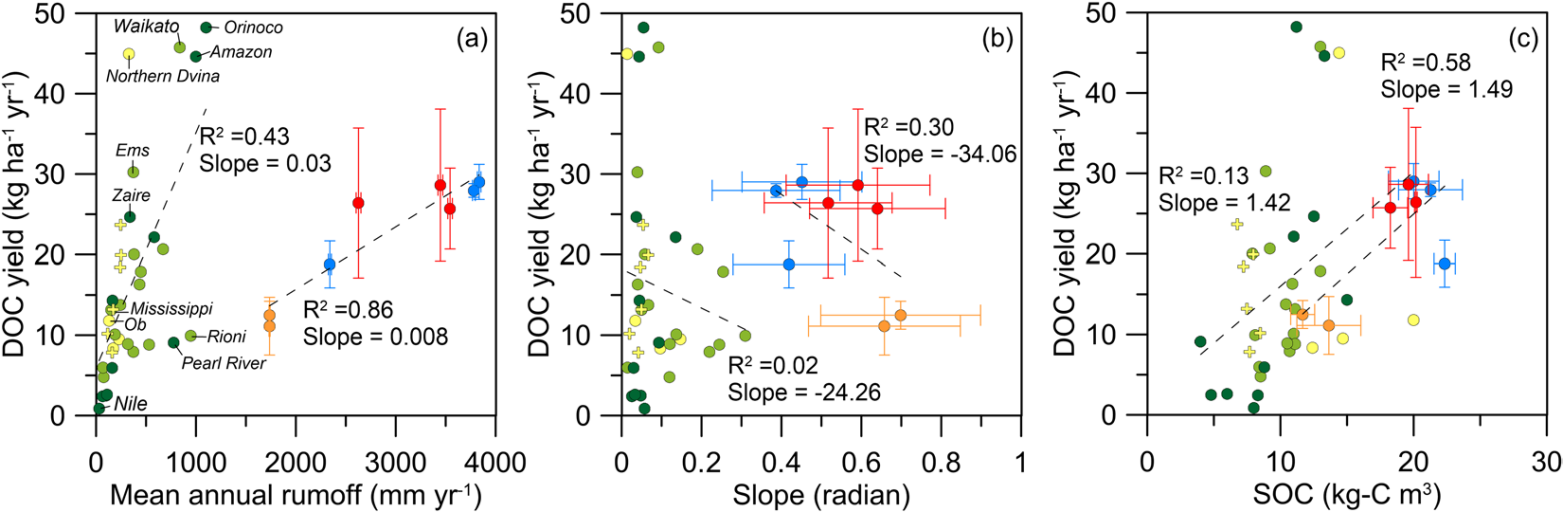
Relationship between DOC yield and mean annual runoff (**a**), mean surface slope (**b**) and soil organic carbon (**c**) of the worldwide rivers from Ludwig, *et al*.^12^ and our SMRs: Chi-Chia-Wan (red), Li-Wu (orange), and Bei-Shi (blue) River. The data from Ludwig, *et al*.^12^ were divided into three biome types: tropical (dark green), temperate (light green), and tundra and taiga (yellow). Additionally, the DOC yields of Arctic rivers (yellow cross) were from Guo, *et al*.^34^, Kaiser, *et al*.^33^, and Raymond, *et al*.^25^.

A comparison of Ludwig’s rivers and our cases on the relationship between DOC yield and runoff, slope and SOC was shown in Fig. 5. Additionally, we introduced several independent studies from Arctic rivers, where climate and environmental changes are having profound impacts on this region^25,33,34^. The SOC in this region varies between 3.4–55.1 kg-C m^−3^ from mountains to lowlands^37^. The figure shows two distinct groups, our SMRs and other rivers. Our annual runoff are larger and the surface slope steeper than those of other rivers while the SOC stocks are comparable. The regressive slopes of the two groups show that runoff is positively correlated with DOC yield for both groups, in which Taiwan SMRs demonstrate more significant correlation (R^2^ = 0.86) (Fig. 5a). Interestingly, the SMR group would shift to the other group compatibly, if we exclude the influence of typhoon events which account for ~30% of annual runoff and ~25% of DOC yield^20^. This could shed lights on the change of DOC export by rainfall intensification via warming condition. On the other hand, with regard to the terrain slope, both groups show a negative correlation with DOC yields (Fig. 5b). As for SOC, the positive and similar regressive slopes of the two groups imply that the influence of SOC on DOC yield is universal (Fig. 5c).

## Conceptual model of DOC export in Taiwan

**Figure 6:**
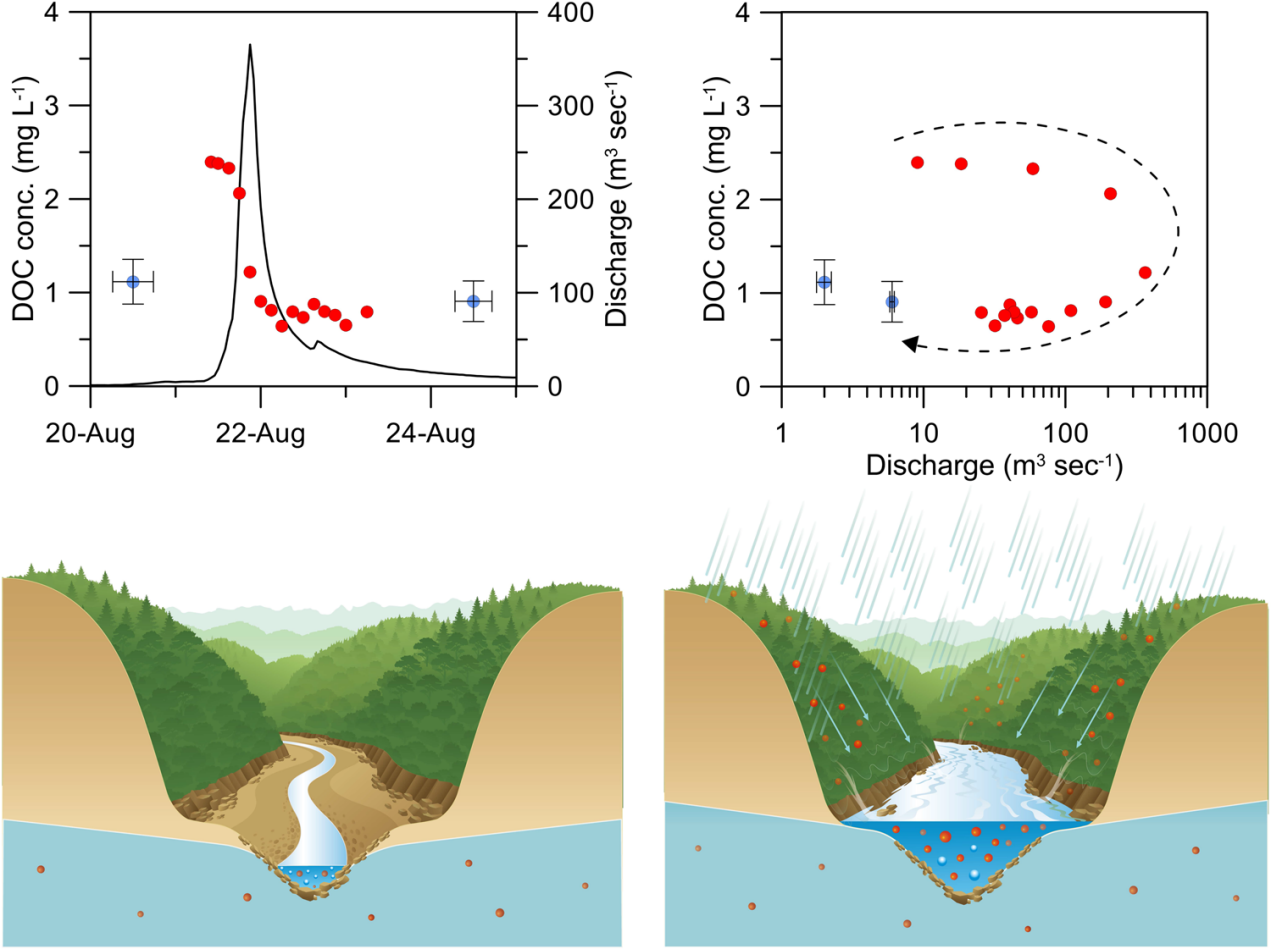
DOC concentration against discharge during typhoon period. The red and blue dot represent the DOC samples during typhoon and normal flow regime (upper panel). Note the error bars of blue points indicate the samples are taken before and after the invasion of typhoon within 20 days. The red and blue dots in lower panel indicate the allochthonous and autochthonous DOC.

Generally, DOC in stream fuels bacterial production and stimulates aquatic productivity of multiple trophic levels^38^. However, the autochthonous DOC (produced by autotrophs within the river system) accumulation in SMRs of Taiwan might be inhibited by fast flows and limited channel volume, even in the growing season. Thus, the mean DOC concentration is quite low (Fig. 6). For example, the DOC concentrations from the groundwater in B1 (unpublished data) is 0.47–0.61 mg-C L^−1^, almost equivalent to the average of stream water. As a rainstorm is amplifying, terrestrial materials containing a considerable amount of DOC are increasingly flushed out from the riparian zone and reach the apex before the storm reaches its peak. During the rising limb of the hydrograph, the surging surface runoff influxes into the stream carrying high amounts of dissolved and suspended organic matter. In this regard, the allochthonous DOC (imported from the watershed) is likely transported by surface runoff into the stream. In fact, Schmidt, *et al*.^39^ investigated the DOC and DON in the forest ecosystem in Chi-Lan Mountain (located between Chi-Chia-Wan, Li-Wu, and Bei-Shi). They found that the DOC concentration in the forest floor and the near-surface seepage could be up to 28.4 and 12.3 mg L^−1^, respectively. Thus, the contribution of the allochthonous DOC from top soil to rivers is seen as a major source, when the hydrological connectivity is increased to link different landscape units during the storm periods^40^. Meanwhile, during typhoon events, floods frequently reshape the channel morphology by turbulence and abrasion phenomena^41^. Following the falling limb of the hydrograph, the deposited sediment and remnant surface runoff (enriched allochthonous DOC) may provide the basis to initiate new ecologic successions and rebuild the benthic microbial communities. The effect of rapid evolution of new succession in SMRs on DOC transport (as well as other nutrients) needs further exploration.

## Conclusions

Conceptually, DOC generation and export depends on three factors: (1) DOM availability and DOC quality (autochthonous and allochthonous DOC), (2) bacterial decomposition rate, and (3) physio-geographic conditions. For watershed scale, physio-geographic factors (runoff, slope and SOC) which regulate DOM availability and substrate transport are effective predictors for estimating DOC transport at annual and regional scale. Besides, our observations show that the annual mean DOC concentration at the eight sites is ~0.78 mg L^−1^, which is much lower than the global average of 5.29 mg L^−1^. By contrast, the DOC yields with an average of 22.51 kg-C ha^−1^ yr^−1^, is higher than the global average of 14.4–19.3 kg-C ha^−1^ yr^−1^. Applying our dataset onto the regressive model proposed by Ludwig, *et al*.^12^, we found significant over-estimation while estimations from Aitkenhead-Peterson and McDowell^13^ showed distinct under-estimation. Although the runoff, slope and SOC are also effective factors, the large biases specified the unusual DOC export behaviors in SMRs. Our elevation-dependent biome (deciduous and warm conifer forests) has the similar level of SOC with temperate forests. However, the steep landscape featuring short residence time and limited channel volume impede the production and accumulation of DOC. But the abundant runoff flushing considerable DOC from watershed elevate the DOC export in SMRs. Meanwhile, the clockwise hysteresis during typhoon events implies that DOC comes from a limited storage in the riparian zone or downslopes supporting the flushing hypothesis. In addition, a proposed conceptual model illustrated the alternating nature of allochthonous and autochthonous DOC transport during typhoon periods. When rainstorms institute the connection from the hillslope to the stream, the riparian zone or downslope area play an important role in transporting terrestrial DOC to streams. Differentiation of the DOC sources should be explored further to link relevant sources of DOC and its transport from terrestrial to the aquatic ecosystems. Notably, rainstorm-induced floods in mountainous area would initiate new succession of aquatic ecosystems and the rapid evolution of the new succession characterizes the distinctive feature of DOC transport in SMRs. This study puts another piece to the puzzle of DOC characteristics in SMRs and provides a fundamental basis for planning future investigations.

## Supplementary information

Supplementary Material
