# Supplementary material for: Unusual Roles of Discharge, Slope and SOC in DOC Transport in Small Mountainous Rivers, Taiwan
Source: Sci Rep. 2019 Feb 7;9:1574. doi: 10.1038/s41598-018-38276-x (PMC6367504; doi:10.1038/s41598-018-38276-x)
Supplement: Supplementary file 1 — Supplementary Material [file 41598_2018_38276_MOESM1_ESM.docx]

Supplementary Material

Unusual Roles of Discharge, Slope and SOC in DOC Transport in Small Mountainous Rivers, Taiwan

By Li-Chin Lee, Ting-Chang Hsu, Tsung-Yu Lee, Yu-Ting Shih, Chuan-Yao Lin, Shih-Hao Jien, Thomas Hein, Franz Zehetner, Fuh-Kwo Shiah, Jr-Chuan Huang

1. Data Acquisition for Studied SMRs

The patterns of land use and slope gradient for the eight sites in the three SMRs were illustrated in ***Fig. S1***, and the elevation and SOC were displayed in ***Fig. 1*** (main text). The land use coverage was provided by NLSE (National Land Surveying and Mapping Centre, Ministry of the Interior, http://ngis.moi.gov.tw). The island-wide SOC data are a compilation of soil samples (since 1981, accumulated to 14,715 soil samples in 2012) and soil series map based on the ordinary kriging technique using spherical model. The RMSEs of the different soil depth (30cm, 50cm, 100cm, integrated) are 2.82, 3.80, 5.40, and 5.71 ^1^.

The environmental metrics of the eight sites were shown in ***Table 1*** (main text). Generally, the mean annual air temperature increases from Chi-Chia-Wan to Li-Wu and then to Bai-Shi River, reversely following the elevation gradient. The mean slope in Li-Wu and Chi-Chia-Wan River ranges from 0.52 to 0.70 radians, and even in the gentlest Bai-Shi River, the gradient varies from 0.39-0.45 radians, which is still higher than other rivers around the world, emphasizing the steepness in Taiwan mountainous watersheds (***Fig. S1***). Although the landscape is steep, plantation agriculture still can be found in Chi-Chia-Wan (cabbage and fruit trees, mainly apple, pear, and peach) and Bai-Shi River (tea plantations) ^2^, while in the Li-Wu River, cultivation is prohibited abiding by the National Park regulations. The mean SOC content within 100 cm depth for eight sub-watersheds varies from 11.7 to 22.3 kg-C m^-3^, which is comparable with other studies in the tropical forests, ~17.7 kg-C m^-3 3^. In Taiwan, due to frequent mass wasting incidents, soil depth is relatively shallow and the SOC content in Li-Wu River is particularly low.


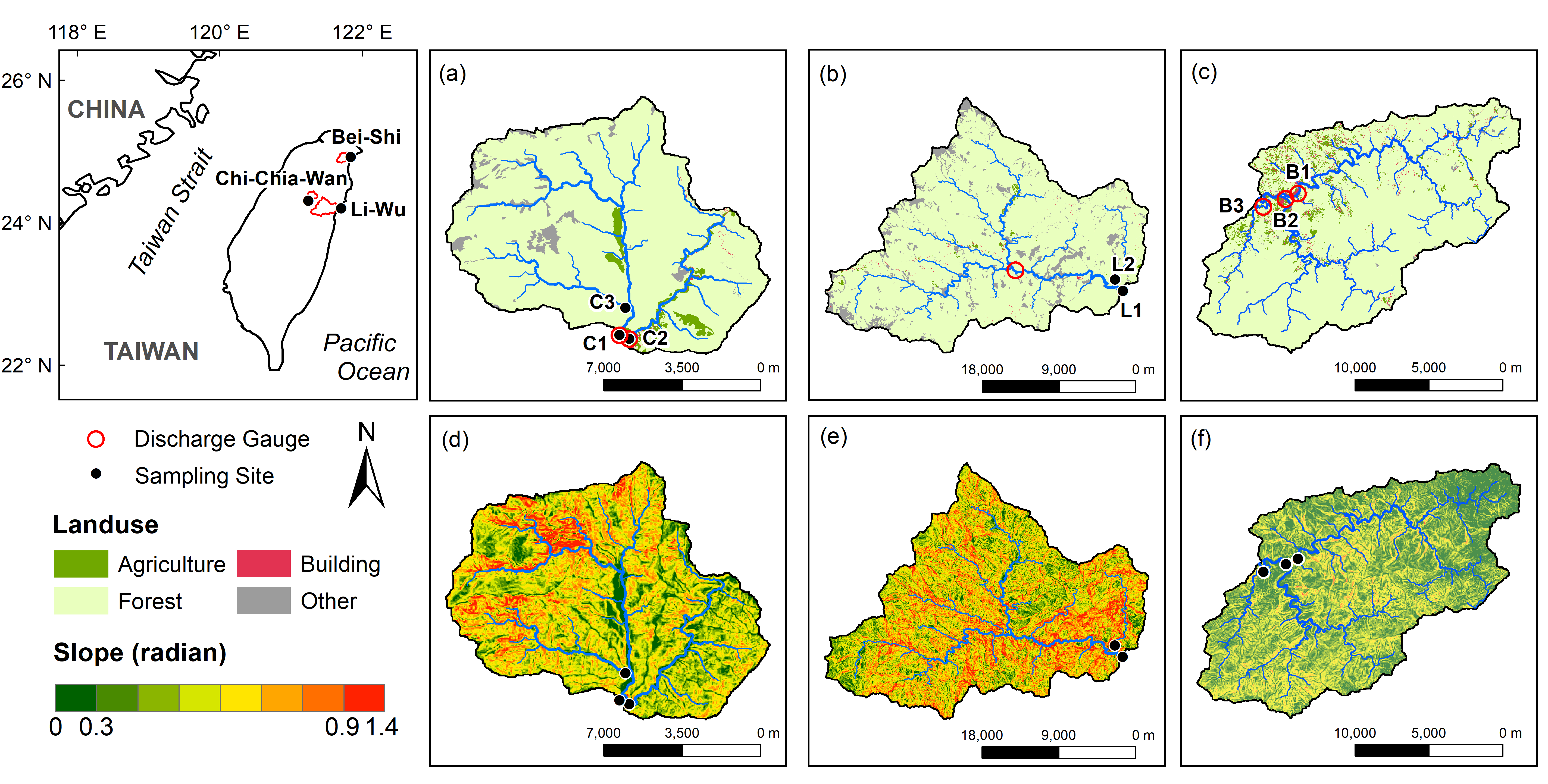


**Figure S1**. The distribution of land use in Chi-Chia-Wan (a), Li-Wu (b) and Bei-Shi (c) River. The distribution of slope in Chi-Chia-Wan (d), Li-Wu (e) and Bei-Shi (f) River.

The six discharge stations maintained by either WRA (Water Resource Agency,) or TPC (Taiwan Power Company) are used in this study. For the ungauged sites (C3 in Chi-Chia-Wan River, and L1 and L2 in Li-Wu River), the discharge was estimated by the area ratio method in which the streamflow was assigned to the values registered at the nearest discharge gauge and then adjusted by the area ratio of the studied site relative to the watershed where the discharge gauge was located ^2,4^. The monthly air temperature and precipitation with a resolution of 5-km were provided by the Taiwan Climate Change Projection and Information Platform project (TCCIP, https://tccip.ncdr.nat.gov.tw/v2/index.aspx). The temperature and rainfall dataset synthesized multiple data sources of more than 300 stations from Central Weather Bureau, WRA, TPC, and Irrigation Association. The synthesized dataset of temperature and precipitation was spatially interpolated by adiabatic lapse rate and inverse distance weighted interpolation, respectively. The detailed data construction and evaluation methods can be found in Weng and Yang ^5^.

2. Water sampling and DOC measurement

Our streamwater samples were collected from 2007 to 2013. The sampling frequency for normal flow regime was approximately 3.5 days in Chi-Chia-Wan River (Jan. 2007-Dec. 2008) and Bai-Shi River (Jan. 2012-Dec. 2013) and around 9 days in Li-Wu River (May 2009-Apr. 2011). Owing to the different sampling period, we checked the hydroclimatic state between different sampling periods and long-term period (2002-2012). The annual averages of air temperature during sampling periods were consistent with the long-term averages, whereas the annual rainfall during sampling periods show a diverse fluctuation since the annual rainfall variability is large. Despite the variation of annual rainfall, the relative annual rainfall of the three SMRs was still consistent with the long-term pattern.

During typhoon events, sampling was performed every 3-hour for the selected typhoons, but not for all typhoons owing to the limited manpower. In most cases, we collected the water samples by 1-L low-density polyethylene (LDPE) and the collected water samples were filtered with pre-combusted (at 450⁰C for 3 hours) Whatman® glass fiber filters GF/F (0.7μm) *in situ*. The filtrates were divided into a pre-combusted (at 450⁰C for 3 hours) 40 ml glass vial and 0.25 ml of concentrated phosphoric acid (85%) was added immediately. Samples were stored in an ice chest during transport until subsequent analysis returning to the laboratory. The DOC is determined using an Analytik Jena multi N/C^®^ 3100 analyzer with a detection limit of 4 μg L^-1^. Acidified water samples were purged with pure oxygen to remove inorganic carbon. Then, the samples were combusted at 950⁰C and the released CO_2_ was measured with a radiation nondispersive infrared (NDIR) detector.

3. DOC Flux estimation

The LOADEST (Load Estimator), a site-specific regression model that utilizes a function of discharge and Julian day to predict the flux ^6^ is applied in this study. This model uses discrete pairs of DOC concentration and instantaneous discharge to determine the ’best’ regression model and the flux ^7^. The general form of the multiple regression can be expressed as the following:

$\mathrm{In}\left( F \right)=a_{0}+\sum_{i=1}^{n} a_{i}X_{i}$ (1)

Where *F* is the estimated flux and *a_i_* is the model coefficient. *X_i_* is an explanatory variable (e.g. discharge) and *n* is the number of explanatory variables. The statistical estimation method, AMLE (Adjusted Maximum Likelihood Estimation), is used to assess the model coefficients. We preliminarily evaluated numerous regressions to our 8 sites by the AMLE and the AIC (Akaike Information Criteria ) for model selection ^8^. After preliminary testing, the following regression model is used:

$\mathrm{In}\left( F \right)=a_{0}+a_{1}\ln\left( Q \right)+a_{2}\ln\left( Q^{2} \right)+a_{3}\sin\left( 2\pi\cdot dtime \right)+a_{4}\cos\left( 2\pi\cdot dtime \right)+a_{5}(dtime)+a_{6}({dtime)}^{2}$ (2)


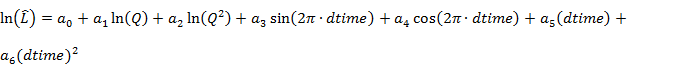

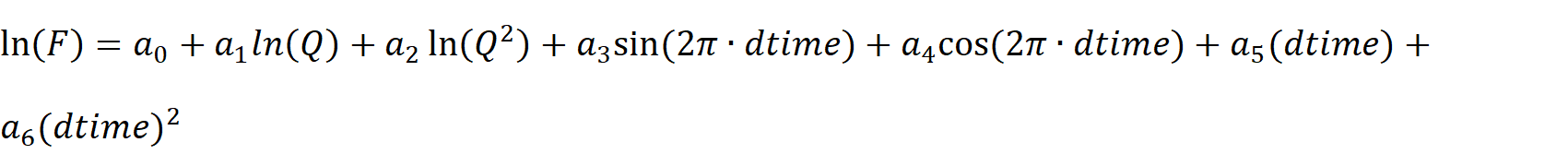


Where *Q* and *dtime* represent the discharge and Julian day (in decimal form), respectively. The coefficients, *a_1_*, and *a_2_*, are discharge associated terms representing the hydrological control. The other coefficients, (*a_3_*, *a_4_*) and (*a_5_*, *a_6_*), are regulated by seasonal oscillations and long-term accumulation or dissipation.

The estimated DOC yields from LOADEST against observations at C1, L1, and B1 were taken as examples and shown in ***Fig. S2***. Note that we used yield (defined as the flux normalized by drainage area, in units of mass per unit area per unit time) instead of flux to make results comparable among sites. Briefly, the estimated DOC yields were in satisfactory agreement with the observations. The Nash-Sutcliffe model efficiency coefficient (NSE, Nash and Sutcliffe ^9^) at the three example sites were 0.94 (for C1), 0.98 (for L1), and 0.89 (for B1), respectively. Besides, the model residuals generally matched the normal distribution, which is requested by the application of AMLE regression. The detailed information on regression coefficients of the eight sites was shown in ***Table S1***. Generally, the NSE values ranged from 0.72 to 0.99 and the Bp varied from -10.82 to 2.31. From the perspective of seasons, the NSE values in wet season were higher than those in dry season, whereas the Bp values between seasons were similar (***Table S2***). Low NSE values in dry season were expected, because the control of streamflow is inept in the low flow.


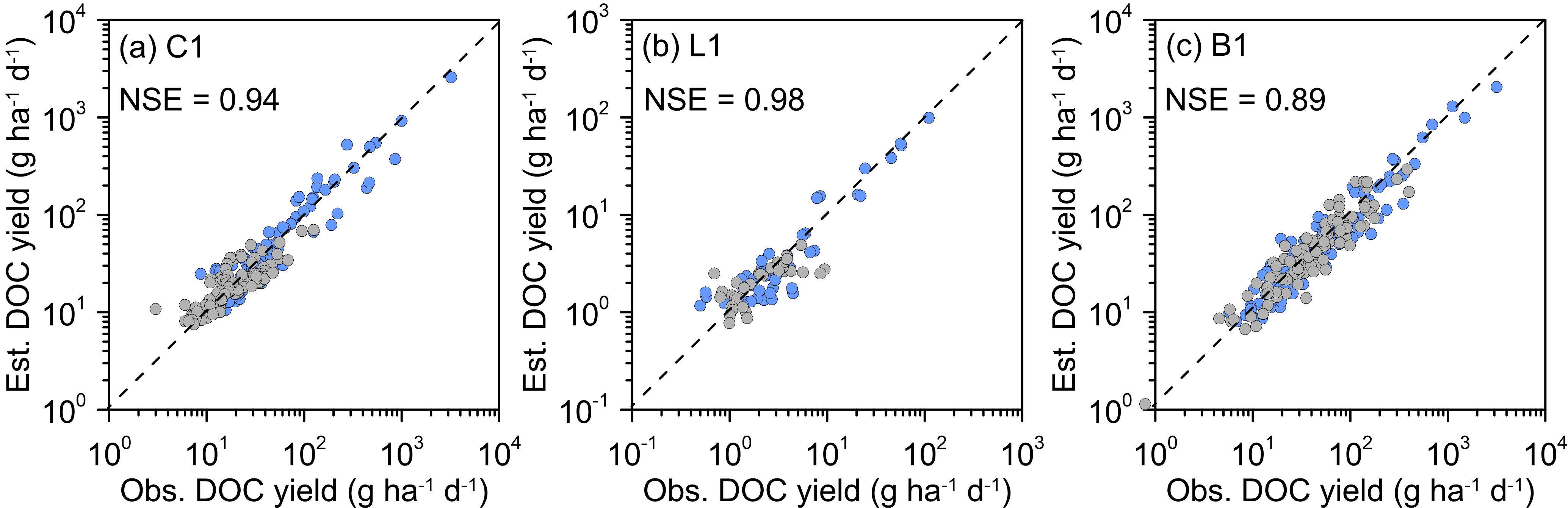


**Figure S2**. Scatter plot of observed and estimated DOC yield for C1 (a), L1 (b), and B1 (c), respectively. The gray and blue dots represent the samples in dry and wet season.

**Table S1.** Regression coefficients, NSE, and Bp at each sampling site.

| site | *a_0_* | *a_1_* | *a_2_* | *a_3_* | *a_4_* | *a_5_* | *a_6_* | NSE | Bp (%)* |
| --- | --- | --- | --- | --- | --- | --- | --- | --- | --- |
| C1 | 6.288 | 0.976 | 0.057 | -0.241 | -0.075 | -0.516 | 0.200 | 0.94 | -4.48 |
| C2 | 4.901 | 1.062 | 0.014 | 0.101 | -0.038 | -0.441 | 0.203 | 0.87 | 1.61 |
| C3 | 4.659 | 1.102 | -0.003 | -0.040 | -0.022 | -0.307 | 0.299 | 0.72 | -10.82 |
| L1 | 8.721 | 1.256 | 0.025 | -0.289 | 0.044 | -0.237 | -0.715 | 0.98 | 2.31 |
| L2 | 6.424 | 1.248 | 0.068 | -0.120 | -0.004 | -0.071 | -0.783 | 0.99 | -0.62 |
| B1 | 6.134 | 0.987 | 0.018 | 0.033 | -0.359 | 0.368 | 0.220 | 0.89 | -3.12 |
| B2 | 4.659 | 1.012 | -0.006 | 0.114 | -0.323 | 0.299 | 0.246 | 0.85 | -7.62 |
| B3 | 2.742 | 0.984 | 0.001 | 0.042 | -0.282 | 0.348 | 0.396 | 0.81 | -5.66 |

*Bp (%), the yield bias in percent, is the estimated minus the observed over the observed.

**Table S2.** The NSE and Bp values for dry and wet season.

| site | Dry season | | Wet season | |
| --- | --- | --- | --- | --- |
|  | NSE | Bp (%)* | NSE | Bp (%)* |
| C1 | 0.66 | -8.83 | 0.93 | -10.69 |
| C2 | 0.86 | -6.48 | 0.88 | -2.67 |
| C3 | 0.86 | -3.47 | 0.71 | -19.11 |
| L1 | 0.30 | -16.56 | 0.98 | -5.39 |
| L2 | 0.52 | -7.24 | 0.99 | -8.27 |
| B1 | 0.70 | -3.79 | 0.87 | -11.60 |
| B2 | 0.46 | -7.16 | 0.84 | -15.31 |
| B3 | 0.89 | 1.22 | 0.79 | -14.76 |

*Bp (%), the load bias in percent, is the estimated minus the observed over the observed.

4. Correlation between DOC yields and environmental factors

The correlation coefficient matrix was performed for investigating the inter-correlation between DOC yields and environmental factors for the eight sites (***Table S3***). The results showed that the DOC yield was significantly and positively correlated with the mean annual runoff and SOC, and was negatively correlated with mean surface slope, which showed a moderate coefficient and considered insignificant.

**Table S3.** The correlation matrix among annual DOC yield and environmental factors for eight sites.

|  | DOC yield | Mean annual Temp. | Mean annual runoff | Mean surface slope | SOC | Pop. density^1^ | Agri.^2^ |
| --- | --- | --- | --- | --- | --- | --- | --- |
| DOC yield (kg-C ha^-1^ yr^-1^) | 1.00 |  |  |  |  |  |  |
| Mean annual Temp. (⁰C) | 0.00 | 1.00 |  |  |  |  |  |
| Mean annual runoff (mm yr^-1^) | 0.93** | 0.10 | 1.00 |  |  |  |  |
| Mean surface slope (Radian) | -0.55 | -0.74* | -0.51 | 1.00 |  |  |  |
| SOC (kg-C m^-3^) | 0.76* | 0.34 | 0.66 | -0.86** | 1.00 |  |  |
| Pop. density (pop/km^-2^) | 0.37 | 0.89** | 0.49 | -0.89** | 0.63 | 1.00 |  |
| Agri. (%) | 0.43 | 0.23 | 0.14 | -0.68 | 0.70 | 0.34 | 1.00 |

^1^ Pop. density indicates the mean population density within the watershed during 2002-2012.

^2^Agri. indicates the proportions of the agricultural land within the watershed.

‘*’ and ‘**’ indicates the statistically significance with p-value < 0.05 and 0.01, respectively, for a two-tailed test.

However, human disturbances (e.g. population, agriculture area and wastewater) and temperature did not show a significant correlation. Theoretically, human disturbances should, in most cases, elevate the DOC yield. For example, the DOC yields in agricultural watersheds in Illinois, USA increase with the fractions of agricultural area, particularly in high flow regimes^10^. The insignificant coefficients of human disturbance are probably due to rather limited population and agricultural activities in our SMRs. Temperature, which is highly associated with enzymatic activities and chemical reaction rates (e.g. Q10 factor), can enhance decomposition rate of soil organic matter, particularly in temperate zones, and consequently influence the DOC accumulation and export^11^. The indistinct seasonality of DOC concentration in our SMRs may imply that the temperature effect is overridden by the abovementioned controlling factors for reason that the steeper slope leads to higher flow velocity and shorter residence time, which may impede DOC accumulation and export. Nevertheless, this correlation coefficient matrix affirms that runoff, slope and SOC are the first-order controlling factors in DOC yields for both the large rivers and SMRs.

**References**

1. Ho, C. P. *Spatial variability of soil organic carbon and related climate and topographical factors in Taiwan* Doctoral dissertation thesis, National Pingtung University of Science and Technology, (2013).

2. Lin, T. C. *et al.* Effects of mountain tea plantations on nutrient cycling at upstream watersheds. *Hydrol Earth Syst Sc.* **19**, 4493-4504; <http://doi.org/10.5194/hess-19-4493-2015> (2015).

3. Jobbagy, E. G. & Jackson, R. B. The vertical distribution of soil organic carbon and its relation to climate and vegetation. *Ecol Appl.* **10**, 423-436; <http://doi.org/10.2307/2641104> (2000).

4. Lee, T. Y., Huang, J. C., Kao, S. J. & Tung, C. P. Temporal variation of nitrate and phosphate transport in headwater catchments: the hydrological controls and land use alteration. *Biogeosciences.* **10**, 2617-2632; <http://doi.org/10.5194/bg-10-2617-2013> (2013).

5. Weng, S. P. & Yang, C. T. The Construction of Monthly Rainfall and Temperature Datasets with 1km Gridded Resolution over Taiwan Area (1960-2009) and Its Application to Climate Projection in the Near Future (2015-2039). *Atmospheric Science.* **40**, 349-369; <http://doi.org/https://dbar.ttfri.narl.org.tw/ATMOS/40-4/40-4-2.pdf> (2012).

6. Runkel, R. L., Crawford, C. G. & Cohn, T. A. Load Estimator (LOADEST): A FORTRAN Program for Estimating Constituent Loads in Streams and Rivers. 69 (2004).

7. Huntington, T. G. & Aiken, G. R. Export of dissolved organic carbon from the Penobscot River basin in north-central Maine. *Journal of Hydrology.* **476**, 244-256; <http://doi.org/10.1016/j.jhydrol.2012.10.039> (2013).

8. Judge, G. G., Hill, R. C., Griffiths, W. E., Lutkepohl, H. & Lee, T. C. *Introduction to the theory and practice of econometrics (2d ed.).* (John Wiley, pp. 1024, 1988).

9. Nash, J. E. & Sutcliffe, J. V. River flow forecasting through conceptual models part I — A discussion of principles. *Journal of Hydrology.* **10**, 282-290; <http://doi.org/10.1016/0022-1694(70)90255-6> (1970).

10. Royer, T. V. & David, M. B. Export of dissolved organic carbon from agricultural streams in Illinois, USA. *Aquat Sci.* **67**, 465-471; <http://doi.org/10.1007/s00027-005-0781-6> (2005).

11. Freeman, C., Ostle, N. & Kang, H. An enzymic 'latch' on a global carbon store. *Nature.* **409**, 149; <http://doi.org/10.1038/35051650> (2001).
